# Single‐center study on SARS‐CoV‐2 infection in patients affected by CML: Vaccination status and bosutinib therapy as possible protective factors for hospitalization

**DOI:** 10.1002/cam4.5923

**Published:** 2023-04-11

**Authors:** Ivan Civettini, Laura Antolini, Filippo Brioschi, Giovanni Paolo Maria Zambrotta, Veronica Guglielmana, Carlo Gambacorti‐Passerini

**Affiliations:** ^1^ Department of Medicine and Surgery University of Milano‐Bicocca Monza Italy; ^2^ Hematology Division San Gerardo Hospital, ASST Monza Monza Italy

**Keywords:** bosutinib, chronic myeloid leukemia, COVID, hematology, myeloproliferative neoplasms, SARS‐CoV‐2

## Abstract

**Introduction:**

COVID‐19 pandemic had a considerable impact among haematological patients. On the other hand, the effect of this disease on patients (pts) affected by Chronic Myeloid Leukemia (CML) is not clearly defined.

**Objectives:**

The primary objective of this study was to evaluate mortality‐hospitalization rates and possible protective factors for hospitalization in CML pts affected by COVID.

**Methods:**

We collected data from CML patients followed at our institution whotested positive for SARS‐CoV‐2 infection. The following variables were assessed: demographical data, type of TKI therapy, vaccination status, presence of cardiovascular disease (CVD), period of infection, COVID‐19 presenting symptoms, severity and mortality. Data were collected retrospectively and then analysed in univariate and multivariate analysis.

**Results:**

Out of a total of 325 CML pts treated at our institution, we recorded 72 SARS‐CoV‐2pts (22%) who tested positive with a SARS‐CoV‐2 PCR assay. Twenty two were infected in 2020 (30%), 16 patients in 2021 (22%) and 34 in 2022 (46%); with a hospitalization rate of 27%, 25% and 3% respectively. Of the 72 confirmed infections, 13 pts (18%; (CI) 10–28) were asymptomatic and 48 (66%; CI: 55–76) had mild symptoms. A total of 11 pts were admitted to hospital and 3 of these required ICU admission. No deaths were recorded. The probability of hospitalization was significantly reduced if patients were vaccinated (odds ratio OR 0.037 with CI: 0–0.33 *p* 0.002) or treated with Bosutinib (OR 0.06 with CI: 0–0.5 *p* 0.008).

**Conclusion:**

In the present study, no significant increase in mortality was noted among patients with CML as compared to the general population inItaly. Vaccination and treatment with bosutinib were identified as baseline characteristics that were associated with a decreased risk of hospitalitazion resulting from COVID‐19 infection.

## Graphical abstract

We collected data from 325 consecutive CML patients followed at our institution, of whom 67 tested positive for SARS‐CoV‐2. We found out that the death rate observed in our serie was not different from that observed in national data. Moreover, the risk of hospitalization was significantly reduced in vaccinated patients or in those taking bosutinib.

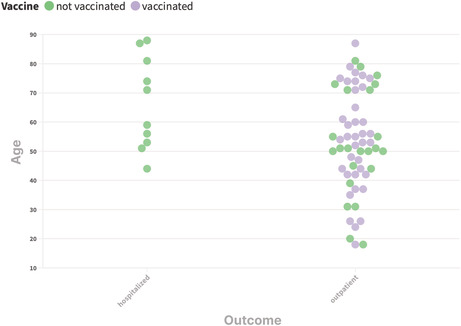

## 1 INTRODUCTION

The advent of the COVID‐19 pandemic had a considerable impact among hematological patients, with high mortality rates as reported by Passamonti et al.^3^

The same authors, however, highlighted that patients with chronic myeloid leukemia (CML) receiving TKI were at a lower risk of mortality compared with other hematological patients. These findings were not confirmed by the CANDID^6^ study in which a 13.7% COVID‐19 mortality rate was reported among CML patients. This value was later reassessed by Breccia et al. who reported a lower rate of 5.5%, but apparently still higher than the general population during the first year of the pandemic (2.9%).^2^ To better understand this aspect, we collected data from 72 of 325 CML patients followed at our institution who tested positive for SARS‐CoV‐2 infection.

## 2 MATERIALS AND METHODS

Consecutive CML patients treated at our institution (San Gerardo Hospital, Monza) and with a PCR‐based confirmed SARS‐CoV‐2 infection diagnosed from February 2020 to October 2022 were followed. The aim of our study was to evaluate the severity and mortality rates of COVID‐19 in CML patients. The following variables were assessed: demographical data, type of TKI therapy or Treatment‐free Remission (TFR), vaccination status, presence of cardiovascular disease (CVD) risk factors (hypertension, hypercholesterolemia and diabetes), of previous cardiovascular events (CVE) or of deep venous thrombosis (DVT), period of infection, COVID‐19 presenting symptoms, severity (hospitalized/admitted to intensive care unit [ICU]), and mortality. Patients were defined vaccinated if they had received at least two doses of SARS‐CoV‐2 vaccine before infection and vaccination was collected as a categorical dichotomous variable. Treatment‐free Remission was defined when patients in stable deep molecular response (DMR) discontinued TKI therapy. Data were collected retrospectively and then analyzed using GraphPad Prism 9 by Dotmatics. Differences in the distribution of categorical variables were evaluated using Fisher's exact test and an exact multiple logistic regression model was used to identify independent predictors of response.

## 3 RESULTS

Of a total of 325 CML patients treated at our institution, we recorded 72 SARS‐CoV‐2 patients (22%) who tested positive with a SARS‐CoV‐2 PCR assay. Female patients were 39 (54%) and male patients were 33 (46%). The median age was 55 years old (Table 1).

**TABLE 1:** Baseline demographic characteristics, TKI therapy, comorbidities and vaccination status at the time of diagnosios of COVID.

	Outpatients	Hospitalized	Total
	*n* 61 (84%)	*n* 11 (16%)	*n* 72
Median age	53	63	55
Age			
Age <50	21 (95%)	1 (5%)	22
Age >50	40 (79%)	10 (21%)	50
Gender			
M	28 (85%)	5 (16%)	33
F	33 (84%)	6 (16%)	39
TKI			
Imatinib	24 (85%)	4 (15%)^a^	28
Bosutinib	21 (100%)	0	21
Nilotinib	0	1 (100%)	1
Ponatinib	1 (50%)	1 (50%)	2
Dasatinib	3 (75%)	1 (33%)	4
TFR	11 (85%)	2 (15%)	13
No TKI	1 (33%)	2 (67%)^b^	3
Vaccine			
Yes	37 (100%)	0	37
No	23 (70%)	10 (30%)	33
Not known	1 (50%)	1 (50%)	2
Cardiovascular risk			
Yes	23 (85%)	4 (16%)	27
No	38 (84%)	7 (16%)	45
Year of infection			
2020	16 (70%)	6 (30%)	22
2021	12 (75%)	4 (25%)	16
2022	33 (97%)	1 (3%)	34

Abbreviation: TFR, Treatment‐free remission.

^a^ One patient admitted to ICU.

^b^ Both patients were admitted to ICU.

No deaths were recorded (0%, 95%, confidence interval (CI): 0%–4.5%).

Of these 72 patients, 28 were on treatment with Imatinib (39%), 21 with Bosutinib (30%), and 13 were in TFR (19%). Dasatinib, Nilotinib, and Ponatinib were, respectively, took by 4, 1, and 2 patients, respectively. Two patients were at CML onset and therefore without treatment, while one patient self‐discontinued therapy without a DMR (NO‐TKI). There were no significant differences among the median ages of patients taking different TKIs (Imatinib 54‐year‐old; Bosutinib 51‐year‐old).

At the time of infection, 27 patients (37%) had CVD risk factors, a previous CVE or a previous DVT. Thirty‐tree patients (46%) were not vaccinated when COVID‐19 was diagnosed, either because of lack of vaccine (before February 2021) or because of personal decision. Of the 37 vaccinated patients, three and two doses were received by, respectively, 19 and 18 patients. Mean ages did not differ significantly between vaccinated and not vaccinated patients (55 vs. 53 years).

Of the 72 confirmed infections, 13 patients (18%; [CI] 10–28) were asymptomatic and 48 (66%; CI: 55–76) had mild symptoms. The median age of these patients was 50‐ and 55‐year‐old, respectively. A total of 11 patients were admitted to hospital and three of these patients required ICU admission; the median age of the hospitalized patients was 63 years. All the three patients admitted to ICU were not vaccinated and two of them were not in active therapy with TKI without a DMR; one patient was at CML onset, the other self‐discontinued therapy.

Twenty‐two patients were infected in 2020 (30%), 16 patients in 2021 (22%), and 34 in 2022 (46%); with an hospitalization rate of 27%, 25%, and 3%, respectively. The three patients admitted to ICU were evenly distributed in 2020, 2021, and 2022.

When analyzing the vaccination rate among patients at different years, in 2022, there was a 85% vaccination rate, 56% in 2021, and 0% in 2020.

When analyzed by univariate analysis the risk of hospitalization was significantly reduced by being vaccinated (0% vs. 30%; *p* < 0.001) and in therapy with bosutinib (0% vs. 23%; *p* 0.014). (Fisher's exact test). In multivariate analysis, the probability of hospitalization was significantly reduced if patients were vaccinated (odds ratio OR 0.037 with CI: 0–0.33; *p* 0.002) or treated with Bosutinib (OR 0.06 with CI: 0–0.5; *p* 0.008). The impact of bosutinib in multivariate analysis was assessed also by comparing bosutinib vs other TKIs therapy and excluding patients in TFR (OR 0.07, CI: 0–0.7; *p* = 0.02).

The probability of hospitalization was not significantly associated with age, either in univariate (>50 years old; 5.0% vs. 20%; *p* 0.15) or in multivariate analyses (OR 0.26 with CI: 0–3; *p* 0.29). Nevertheless, when restricting the analysis to unvaccinated patients, being older than 50 years old was associated with a higher probability of hospitalization in univariate analysis (47% vs. 7%; *p* = 0.021).

CV risk was not associated with a higher probability of hospitalization neither in univariate analysis (*p* > 0.99), nor in multivariate (OR 1.9 with CI: 0.15–26, *p* 0.99).

Even if there was a prevalence of hospitalized patients in 2020 and 2021, when analyzed in multivariated analysis, the year of infection was not associated with a higher risk of hospitalization (2020 vs. 2021; *p* = 0.1 and 2022 vs. 2021; *p* = 0.8).

## 4 CONCLUSIONS

With a follow‐up of 32 months, we registered an infection rate of 22% among patients affected by CML at our center. Most of them were asymptomatic or had mild symptoms and only 11 of 72 required hospitalization, among whom only three were admitted to ICU. No death was recorded.

The death rate reported in our sample, especially when considering the 95% confidence intervals, appeared to be not significantly different from the one reported by a previous Italian study (0%, 95% CI: 0%–4.4% vs 5.5%, 95% CI: 2.9–9.1)^2^ or the one reported for the Italian population (1.1%) from February 2020 to April 2022.^1^ This fact poses the CML population in a position of its own when compared to patients with other hematological malignancies affected by COVID‐19, for whom very high mortality rates were reported.^3^

The apparent excess of mortality observed in previous studies in CML patients^2^;^6^ could be related to a variety of causes rather than to the hematological disease itself; possible explanations could lie in the vaccination status of patients and/or the older age of the analyzed population when comparing the death rates to the general population. Indeed, the impact of age was assessed by previous studies as a predisposing factor for death in CML patients affected by COVID‐19.^2^;^6^ Nevertheless, in our study, we noticed a lack of correlation between severity of infection/hospitalization and age. This could be explained by the vaccination status in our sample (49% vaccination rate) compared with the previous studies, especially, when vaccines were not available. In fact being older than 50 years old did not emerged as a predisposing factor for hospitalization, either in univariate or in multivariate analysis in our sample, unless the analysis was restricted to un vaccinated patients.

The rate of hospitalization in our study (15%) is similar to the one reported by Breccia et al. (17.8%) and apparently lower than the one of the CANDID study (34%). Also this difference could be associated with a different vaccination rate between our sample and the one of previous studies.

When evaluating the year of infection, we noticed that most patients were hospitalized in 2020 and 2021; nevertheless, in multivariate analysis, the year of infection was not related with a different probability of hospitalization. This could be explained by the rates of vaccination through different years; indeed, in 2022, 85% of patients were vaccinated compared to the 56% and 0% of 2021 and 2020, respectively. These result indicate that the vaccination status rather than the year of infection was causally linked to hospitalization.

**FIGURE 1:**
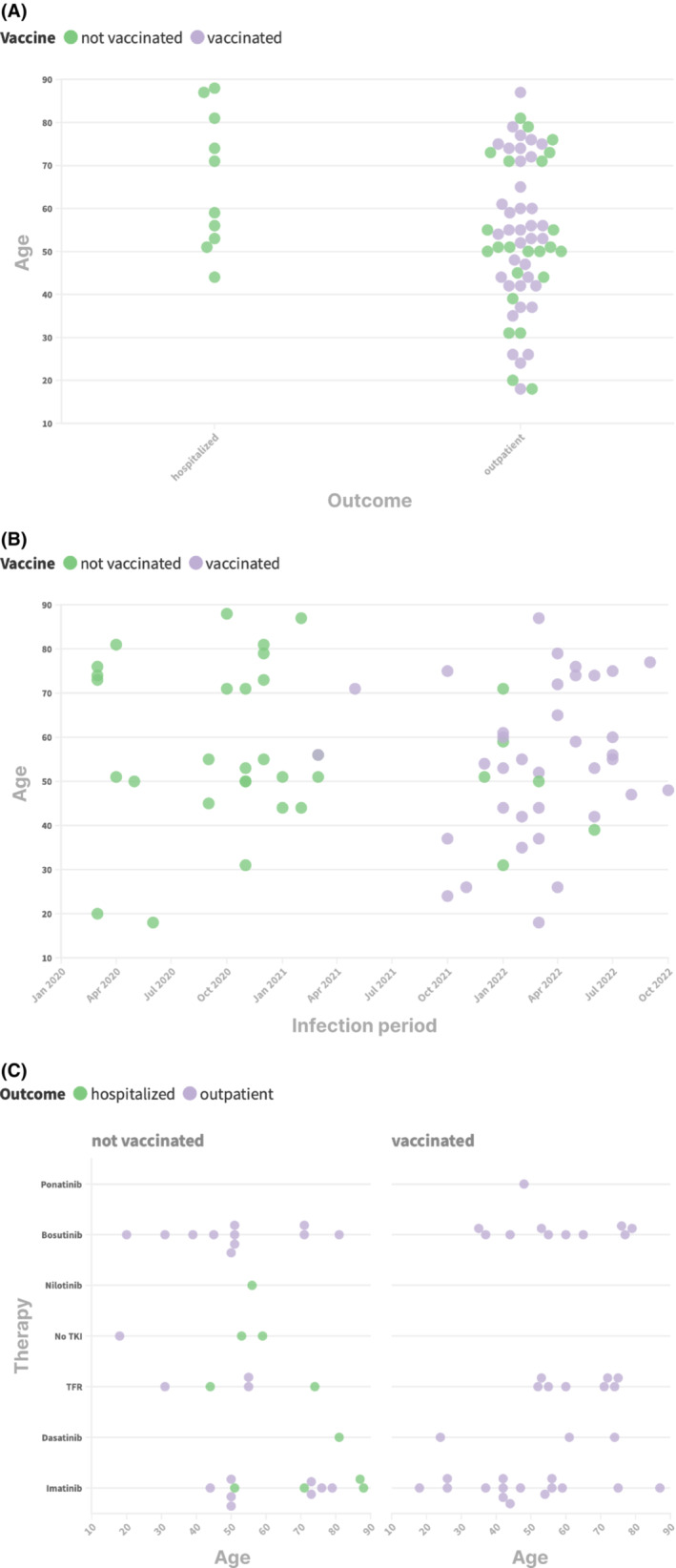
(A) Patients outcome according to age and vaccination status. (B) Vaccination status through different years. (C) Distribution of hospitalized patients according to age, vaccination (yes/no) and therapy. The two patients with unknown vaccination status were omitted from this figure.

When comparing different TKI therapies at the time when COVID‐19 was diagnosed, patients taking Bosutinib appeared to have a reduced probability to be hospitalized compared with patients receiving other TKIs or in TFR, both in univariate and in multivariate analysis (Figure 1). This represents a new results and the potential role of this drug in CML patients affected by COVID‐19 has to be validated and confirmed in future studies. A possible explanation could reside in the inhibitory activity of Bosutinib against the cellular entry activity of SARS‐CoV‐2 reported in a preclinical study^7^ and as an antiviral drug against other viruses such as HHV1,^10^ or in the immunomodulation exerted by bosutinib through its Lyn inhibitory activity.^8^, ^9^

Given the globally negative results of imatinib when used in patients with COVID‐19,^11^ it is likely that the protective role of bosutinib, if confirmed, could therefore reside in its Lyn inhibitory activity rather than in a direct effect on SARS‐CoV‐2, an effect observed at concentrations higher than those achieved in vivo. It has also to be noted that in our study bosutinib was already administered to patients before they became infected, a situation different from patients acutely treated with bosutinib.

In conclusion, in our study, we did not observe an excess of mortality compared with the Italian population and we identified that being vaccinated and possibly on therapy with bosutinib represent baseline characteristics associated with a reduced risk of hospitalization due to COVID‐19 infection.

After all, the fact that CML patients receiving TKI enjoy a normal life expectancy^4^, ^5^ should also be applied to the outcome of the infection with SARS‐CoV‐2.

## AUTHOR CONTRIBUTIONS

**Ivan Civettini:** Conceptualization (equal); data curation (equal); investigation (equal); project administration (equal); resources (equal). **Laura Antolini:** Data curation (equal); formal analysis (equal); methodology (equal); supervision (equal); validation (equal); writing – review and editing (equal). **Filippo Achille Brioschi:** Data curation (supporting); resources (supporting). **Giovanni Paolo Maria Zambrotta:** Data curation (supporting); resources (supporting). **Veronica Guglielmana:** Investigation (supporting); resources (supporting). **Carlo Gambacorti‐Passerini:** Conceptualization (equal); data curation (equal); project administration (equal); supervision (equal); writing – original draft (equal); writing – review and editing (equal).

## CONFLICT OF INTEREST STATEMENT

The authors declare no potential conflict of interests.

## ETHIC APPROVAL STATEMENT

This study was approved by the Ethical Committee.

## Data Availability

The data that support the findings of this study are available from the corresponding author, I.C., upon request.
